# *Fervidobacterium pennivorans* subsp. *keratinolyticus* subsp. nov., a Novel Feather-Degrading Anaerobic Thermophile

**DOI:** 10.3390/microorganisms11010022

**Published:** 2022-12-21

**Authors:** Rubén Javier-Lopez, Edoardo Mandolini, Munavvara Dzhuraeva, Khursheda Bobodzhanova, Nils-Kåre Birkeland

**Affiliations:** 1 Department of Biological Sciences, University of Bergen, N-5020 Bergen, Norway; 2 The Biotechnology Center, Tajik National University, Dushanbe 734025, Tajikistan

**Keywords:** thermophile, keratin degrading, anaerobe, feather degrading

## Abstract

*Fervidobacterium pennivorans* subsp. *keratinolyticus* subsp. nov. strain T was isolated from a terrestrial, high-altitude hot spring in Tajikistan. This strain is an obligate anaerobic rod and their cells occur singly, in pairs, or as short chains under the optimal growth conditions of a temperature of 65 °C and pH 6.5, with peptone, glucose, and galactose as the preferred substrates. The minimum generation time of this strain is 150 min. Strain T can efficiently degrade feather keratin at 65–75 °C; this unusual feature is also exhibited by a few other members of the *Fervidobacterium* genus. The total genome size of this bacterial strain is 2,002,515 base pairs, with a C + G content of 39.0%. The maximum digital DNA–DNA hybridization (dDDH) value of 76.9% was observed on comparing the genome of this strain with that of *Fervidobacterium pennivorans* type strain DSM9078. This study describes the physiological and genomic properties of strain T, with an emphasis on its keratinolytic power and differences from other members of the genus *Fervidobacterium*.

## 1. Introduction

The phylum Thermotogae includes extremely thermophilic anaerobic bacteria, with optimum growth temperatures up to 80 °C [1]. They represent one of the deepest bacterial phylogenetic branches based on a 16S rRNA gene sequence analysis. The physiological characteristics of the members of this phylum are similar to those of the early microbes, who were subjected to extreme environmental conditions during the early stages of life on Earth [2]. All members of Thermotogae are Gram-negative rod-shaped cells surrounded by a characteristic sheath-like envelope or “toga” [3,4]. These bacteria have a fermentative metabolism and have been found in a variety of geothermal environments such as oil reservoirs, hydrothermal vents, and terrestrial hot springs [5]. Since the discovery of the first species in the genus, *Fervidobacterium* [6], six more species of this genus have been isolated from terrestrial hot springs worldwide [7,8,9,10,11,12]. Representatives of the genus *Fervidobacterium* can grow on a range of complex substrates such as cellulose, starch, or proteinaceous compounds; however, only a few members have been described as being capable of breaking down keratin, including *Fervidobacterium islandicum* strain AW-1, *Fervidobacterium thailandense*, and *Fervidobacterium pennivorans* [9,10,11]. A total of 287 Thermotogae genome sequences, including metagenome assemblies, are currently publicly available in the GenBank database (https://www.ncbi.nlm.nih.gov/data-hub/taxonomy/200918/; accessed on 2 August 2022), thereby representing a huge resource of hyper-stable enzymes with potential biotechnological applications.

Keratin is a structural protein present in feathers, hair, skin, wool, horns, etc. and is one of the most abundant polymers on Earth after cellulose and chitin [13]. The presence of a large number of intramolecular cysteine disulfide bonds and inter- and intra-molecular polar (i.e., hydrogen and ionic bonds) and nonpolar bonds (i.e., hydrophobic interactions) makes keratin an extremely recalcitrant protein [14,15]. Keratin is insoluble in water and resistant to weakly acidic or alkaline conditions. It is also resistant to common proteolytic enzymes such as pepsin and trypsin [16]. Different types of keratins exist in nature, and they are characterized by their secondary structure and sulfur content [15,17]. According to their secondary structure, keratins are categorized into alpha and beta keratins. While alpha keratins are present in the mammalian epidermis (e.g., hair, wool, bristles, etc.), beta keratins are found in reptile scales and feathers [18,19]. The most abundant amino acids in feather keratin are serine, cysteine, glutamine, and proline [20]. The high cysteine content in feather keratin contributes to the abundance of disulfide bonds, which stabilize the main structure of the feather and impart robustness and resistance to proteolysis and chemical destruction [19,21]. The main sources of keratin waste are feathers produced during poultry farming and wool production. It has been estimated that more than 2 million tons of wool and 20,000 tons of feathers are produced annually worldwide, which raises environmental concerns [9]. Keratin-laden tissues represent a major challenge in the animal rendering industry. Two-thirds of animal products are discarded because of undigested keratin-containing compounds. Keratin-laden biowaste is generally hydrolyzed by mechanical or chemical treatments to obtain feedstock, fertilizers, glues, or foils [9,16]; however, keratin is only partially degraded by these processes, which are inefficient and expensive. Furthermore, most of the essential amino acids that could be recovered (e.g., serine, cysteine, and proline) are lost and wasted [22]. The current solution to process the huge amount of keratin-based biowaste is to bury it in landfills or incinerate it. In this context, the metabolic versatility of the *Fervidobacterium* group, their thermophilic features, and the increasing availability of thermostable proteases for the degradation of proteinaceous biowaste from agriculture and fisheries have immense potential for biotechnological applications [23,24]. In this study, we describe a new *Fervidobacterium pennivorans* subspecies recovered from a hot spring in Tajikistan, which effectively breaks down native chicken feathers under anaerobic conditions at temperatures up to 75 °C.

## 2. Materials and Methods

### 2.1. Sampling and Cultivation

The sample was collected from the Khodja-Obi-Garm geothermal field, located on the Bank of the Varzob River, 50 km North of Dushanbe, Tajikistan, at 38°53′41.45″ N, 68°47′15.64″ E, at an altitude of 1800 m. The temperature and pH of the spring water were 93 °C and 8.5, respectively, with a conductivity of 4378.3 µS/cm [25]. Water samples were placed in 50 mL tightly sealed anaerobic serum flasks and transported to the laboratory at ambient temperature.

Anaerobic enrichment cultures were prepared according to a modified Hungate technique [26]. The mineral medium (MMF) used for the enrichment and cultivation was composed of the following ingredients (per liter): NaCl, 1 g; MgSO_4_·7H_2_O, 0.3 g; KCl, 0.3 g; NH_4_Cl, 0.5 g; CaCl_2_·2H_2_O, 0.1 g; and KH_2_PO_4_, 0.3 g. A trace element solution (1 mL) was also added, containing (per liter): HCl (25%), 10 mL; FeCl_2_·4H_2_O, 1.5 g; CoCl_2_·6H_2_O, 190 mg; MnCl_2_·H_2_O, 100 mg; ZnCl_2_, 70 mg; Na_2_MoO_4_·2H_2_O, 36 mg; NiCl_2_·6H_2_O, 24 mg; H_3_BO_3_, 6 mg; and CuCl_2_·H_2_O, 2 mg. Finally, 0.5 mL of resazurin (0.2%) was added to the medium as a redox indicator. After sterilization at 121 °C for 20 min and cooling to approximately 60 °C under continuous flushing with nitrogen gas, 10 mL of the vitamin solution was added, which contained the following ingredients: 4-aminobenzoic acid, 8 mg/L; D(+) biotin, 2 mg/L; nicotinic acid, 20 mg/L; Ca-D(+) pantothenic acid, 10 mg/L; pyridoxamine·2HCl, 30 mg/L; thiamine dichloride, 20 mg/L; and vitamin B12, 10 mg/L. Finally, 25% HCl-cysteine (2 mL) was added, and the pH was adjusted to 7.0 with 1 M HCl. The medium was transferred to 50 or 100 mL serum flasks using the Hungate technique, closed with butyl rubber stoppers, crimped with metal seals [26], and subsequently supplemented with 0.5% peptone and 0.05% yeast extract. The headspace contained nitrogen gas. Following inoculation, the cultures were incubated at 65 °C.

To obtain pure cultures, ten-fold serial dilutions for extinction experiments were carried out, yielding a bacterial isolate termed strain T. A battery of eight flasks filled with 9 mL of MMF medium supplemented with 0.5% peptone and 0.05% yeast extract was prepared. One mL of the original culture was transferred to one of these new flasks to make a 10^−1^ dilution; from this one, a ten-fold dilution series up to 10^−9^ was prepared. All the flasks were incubated at 65 °C for 48 h and the process was repeated using inoculum from the highest dilution that yielded growth. The most diluted growth-yielding culture was examined by phase contrast microscopy and appeared to be pure, and this was used as a stock culture for all the following experiments. The strain was stored at 4 °C, and fresh cultures were prepared by inoculating 1 mL of inoculum from the stock culture into 30 mL of an anoxic MMF-based medium as described above. The standard incubation temperature was 65 °C. Additional yeast extract (0.1%) was used for determining the temperature and pH-dependency and when denser cultures were needed.

### 2.2. Microscopy

A phase-contrast microscope (Nikon Eclipse E400, Guangzhou, China) equipped with an oil immersion lens (100×) and a Nikon DS-Fi3 camera was used to observe the bacterial cells and obtain photomicrographs. Scanning electron microscopy (SEM) was performed at the Molecular Imaging Center (MIC) (https://www.uib.no/ en/rg/mic) (accessed on 11 October 2022) and the Electron Microscopy Laboratory (ELMILab) of the University of Bergen (https://www.uib.no/en/geo/111662/scanning-electron-microscope) (accessed on 11 October 2022) using a JEOL JSM-7400F (Tokyo, Japan) and Zeiss Supra 55VP scanning electron microscope (Oberkochen, Germany), respectively. The samples were fixed in 2.5% glutaraldehyde (diluted in the culture media) and stored at 4 °C for 24 h until further processed, as described previously [27].

### 2.3. Physiological Characterization

The strain was tested for growth under strict anoxic conditions on the following substrates: sucrose, lactose, arabinose, galactose, mannose, sorbitol, mannitol, starch, CM-cellulose, glucose, and peptone using an MMF medium supplemented with 0.05% yeast extract. After 24 and 48 h of incubation at 65 °C, the growth was determined using phase-contrast microscopy. Doubling of the cell density after 24 or 48 h was scored as positive or slow growth, respectively. To determine the temperature range and optimal temperature for growth, the bacterial strain was subjected to 38, 55, 65, 70, 75 and 80 °C and tested in duplicates using the MMF medium supplemented with peptone (0.5%) and yeast extract (0.1%). A growth curve was prepared by measuring the optical density of the strain at 600 nm using a UV MIN 1240 Shimadzu spectrophotometer. The cells were grown in batch cultures in 50 or 100 mL serum flasks containing 30 mL of MMF supplemented with 0.5% peptone and 0.05% yeast extract. One milliliter aliquots from a freshly inoculated culture incubated at 65 °C were taken every two hours and centrifuged at 12,000× *g* for 7 min to get rid of the resazurin. The supernatant was discarded, and the cell pellet was resuspended in 1 mL of phosphate buffered saline (PBS) containing (per liter) Na_2_HPO_4_ (2.5 g), NaCl (8 g), KCl (0.2 g), and KH_2_PO_4_ (0.2 g) prior to an *OD* measurement. PBS was used as the blank. All the measurements were performed in triplicates. The generation time (*g*) was calculated from the exponential part of the growth curve using the equation:

$$g=\frac{\ln^{2}}{r}$$

where *r* is the growth rate of the organism, calculated using the equation:

$$r=\frac{\ln\left(OD_{2}/OD_{1}\right)}{t_{2}-t_{1}}$$

where *OD*_2_ and *OD*_1_ are the optical densities measured at times *t*_2_ and *t*_1_, respectively.

### 2.4. 16S rRNA Gene Sequence Analysis

Genomic DNA was extracted and purified from cell pellets using the GenElute^TM^ Bacterial Genomic DNA Kit (Sigma-Aldrich, St. Louis, MO, USA) according to the manufacturer’s guidelines. The 16S rRNA gene was amplified using the primers 27F (5′-GAGTTTGATCCTGGCTCAG) and 1525R (5′-GAAAGGAGGTGATCCAGCC) [28]. The PCR program recommended for Taq DNA Polymerase (BioLabs, Durham, NC, USA) was used, and the PCR products were sequenced using Sanger sequencing [29]. The 16S rRNA gene sequence of strain T was aligned to the 16S rRNA gene sequences of eight members of the genus *Fervidobacterium* including the type strains of the six described species using the ClustalW tool [30]. A phylogenetic tree was constructed using the Neighbor-Joining [31] algorithm of the Mega11 software suite [32,33]. The nucleotide sequence distance was measured using the maximum composite likelihood method [34]. The tree was tested by bootstrapping using 1000 replicates [35]. The 16S rRNA gene sequence of *Thermosipho africanus* was used as the outgroup.

### 2.5. Genome Sequencing and Phylogenomics

Genomic DNA from strain T was extracted using the GenElute^TM^ Bacterial Genomic DNA Kit (Sigma-Aldrich) following the manufacturer’s guidelines. The genomic DNA was sequenced using PacBio technology at Eurofins Genomics, Constance, Baden, Germany (https://eurofinsgenomics.eu/) (accessed on 11 October 2022). Processing of the reads and de novo assembly were performed using CLC Genomic Workbench version 21 (QIAGEN Bioinformatics, Redwood City, CA, USA), which yielded a complete genome sequence of 2,002,515 bases with a coverage of 254×. The genome sequence was submitted to the NCBI database (accession No. CP050868) and annotated using an automatic NCBI annotation pipeline. A genome-based phylogenetic analysis was conducted using the Type (Strain) Genome Server (TYGS) available at the DSMZ website (https://tygs.dsmz.de) (accessed on 11 October 2022) [36] and the Ortho Average Nucleotide Identity (ANI) algorithm [37].

### 2.6. Keratinase Activity Test

The keratinolytic activity was assessed using native chicken breast feathers as the keratin-based substrates. The feathers were washed in a methanol: ethanol solution (1:1) before autoclaving (121 °C, 20 min). Then, 15 ± 5 mg of the feathers was added to 30 mL of MMF medium supplemented with yeast extract (0.05%), to which a 1 mL inoculum of the bacterium was added. An uninoculated flask containing only medium with feather was used as the negative control. The cultures were incubated anaerobically at 70 °C. Every 24 h, the flasks were visually inspected to check for changes in the feather integrity.

## 3. Results

### 3.1. Microscopy and Morphology

**Figure 1:**
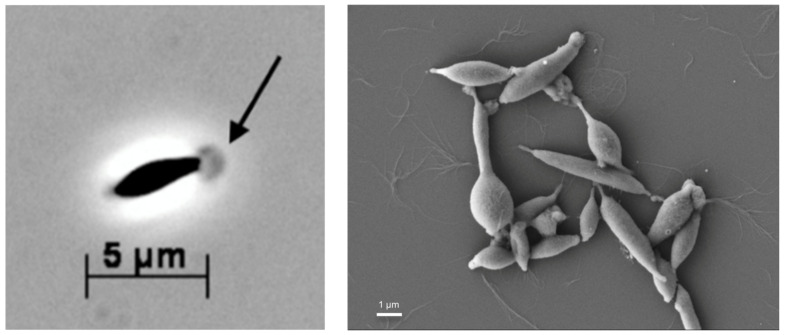
Photomicrographs of strain T cells taken by a phase-contrast microscope (left) and a scanning electron microscope (right). The arrow points to the globular structure of the toga at the termini of the cells. The bars indicate the size in µm.

Following two dilution-to-extinction series in the MMF medium supplemented with peptone, an apparently pure culture was obtained, and termed strain T, with a cellular morphology like other members of the Thermotogales: straight to slightly curved cells (around 1.5 µm × 5 µm) with a membranous sheath-like toga. A spheroid extension of the toga was observed at the end of the cells and was visible under a phase-contrast and scanning electron microscope (Figure 1). Strain T-cells occurred mainly as single entities, although couples or short chains were also common. In addition, some cell aggregates were observed. Spores were not observed. After reaching a stationary phase, many round or irregular translucent cells were observed, indicating cell lysis.

### 3.2. Phylogenetic Identification

**Figure 2:**
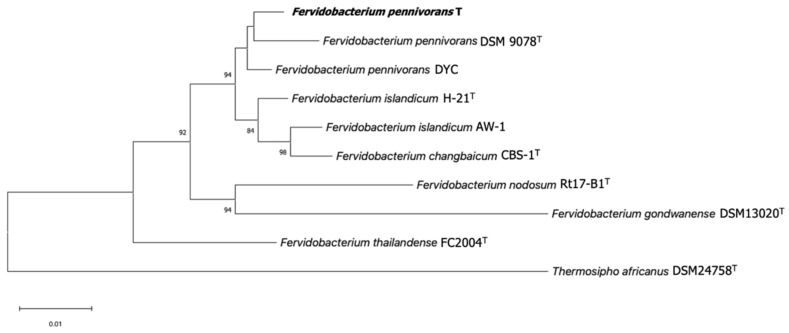
Neighbor-Joining phylogenetic tree of 16S rRNA gene sequences of members of the genus *Fervidobacterium* showing the relationship between strain T (in bold) and other representative strains in this genus. The 16S rRNA gene sequence of *Thermosipho africanus* was used as an outgroup. Bootstrap values as a percentage of 1000 replications are presented at nodes. Bar: 0.01 changes per nucleotide position. Accession numbers: *F. pennivorans* T (ON652409), *Fervidobacterium pennivorans* DSM 9078 (HE582749.1), *Fervidobacterium pennivorans* DYC (NZ_CP011393.1), *Fervidobacterium islandicum* H-21 (M59176.2), *Fervidobacterium islandicum* AW-1 (AF434670.1), *Fervidobacterium changbaicum* CBS-1 (AY878719.2), *Fervidobacterium nodosum* Rt17-B1 (M59177.1), *Fervidobacterium gondwanense* DSM 13020 (Z49117.1), *Fervidobacterium thailandense* FC2004 (JF339226.1), and *Thermosipho africanus* DSM 24758 (DQ647058.1). All ambiguous positions were removed. There were a total of 1285 positions in the final dataset.

A Neighbor-Joining phylogenetic analysis of the 16S rRNA gene sequence of strain T showed that it belongs to the genus *Fervidobacterium*, with a 98.88% sequence identity to the 16S rRNA gene sequence of *Fervidobacterium pennivorans* DSM 9078^T^ (CP003260.1). A phylogenetic tree based on the 16S rRNA gene sequence was constructed for strain T and other members of the genus *Fervidobacterium* (Figure 2). According to the branching order, *F. pennivorans* strain T was placed between *F. pennivorans* type strain (DSM 9078) and *F. pennivorans* strain DYC. A maximum-likelihood [38,39] tree confirmed this branching order (Figure S1).

### 3.3. Physiology

A low concentration of yeast extract (0.05%) was essential for the growth of strain T. The strain clearly possessed a heterotrophic metabolism with a preference for carbohydrates such as lactose, galactose, glucose, and sorbitol, as well as protein-derived substrates such as peptone (Table 1). It also grew well on CM-cellulose, but slowly on sucrose, mannose, or starch. No growth was observed when arabinose or mannitol was used as the substrate. Table 1 shows a physiological comparison of all the described members of the genus *Fervidobacterium*. *F. pennivorans* strain T can degrade native chicken feathers at temperatures up to 70 °C, a characteristic that is also exhibited by other fervidobacteria, except *F. nodosum* and *F. gondwanense*.

All the tested strain T cultures were capable of growing on peptone and glucose. Strain T grew within a narrow pH range (6.0 to 7.5 when tested in 0.5 pH increments from pH 5 to 8, with an optimum pH of 6.5). Growth was not observed at NaCl concentrations higher than 3%, with an optimal salt concentration of 1%. Strain T grew within a temperature range of 55 to 75 °C, with an optimal temperature of 65 °C. Growth was not observed at 50 or 80 °C.

The growth curve of *F. pennivorans* strain T was measured under conditions of 65 °C in MMF supplemented with 0.5% peptone and 0.1% yeast extract (Figure S2). With a fresh inoculum of cells, the lag phase lasted approximately 2 h. The logarithmic phase spanned 11 h, with an estimated minimal generation time of 150 min. After 13 h, the cultures entered a long-lasting stationary phase, never exceeding an *OD* of 0.2. After 24 h of incubation, the culture flasks did not lose turbidity, and no death phase was observed after a week of incubation.

### 3.4. Feather Degradation

**Figure 3:**
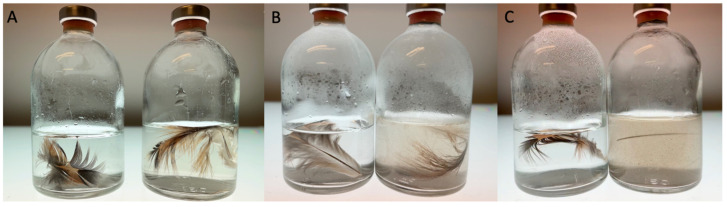
Degradation of native chicken breast feathers by *F. pennivorans* subsp. *keratinolyticus* strain T after 2 days incubation at 70 °C. (**A**–**C**) show cultures at times 0, 24, and 48 h, respectively. The left flask in each panel corresponds to the negative control, containing only mineral medium with feather but without inoculation. Both flasks were held at the same temperature for the same inoculation time.

Strain T degraded native chicken feathers in the MMF medium supplemented with 0.05% yeast extract. The degradation of the breast feathers was almost complete after 48 h of incubation (Figure 3). For wing feathers, which were more robust, degradation started after 7 days of incubation under the same conditions, with a complete degradation within 10 days. Trace amounts of yeast extract were essential for the feather degradation, indicating that feathers cannot serve as the sole carbon and energy source or that yeast extract triggers the keratinolytic activity of the strain.

**Figure 4:**
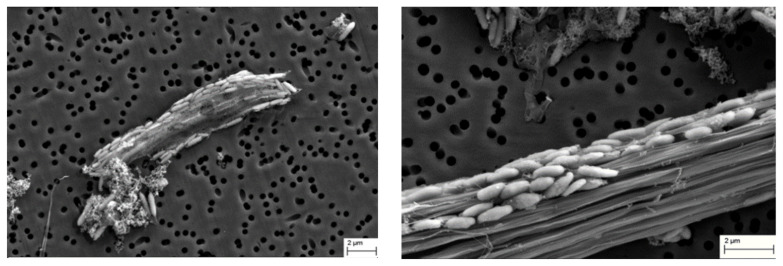
Scanning electron microscope images of strain T grown in the presence of chicken feather. Images were taken after 36 (left) and 48 h (right) of incubation at 70 °C. Bar = 2 µm.

An active feather-degrading culture was examined using scanning electron microscopy. Bacterial cells were found to attach to the keratin fibers (Figure 4), indicating the involvement of cell-bound factors in keratin degradation.

### 3.5. Genome Characteristics and Phylogenomics

The *genome of F. pennivorans* strain T was sequenced using PacBio technology. The complete genome consists of a 2,002,515 bp chromosome, with an average GC-content of 39.0%. By annotation with the NCBI Prokaryotic Genome Annotation Pipeline (PGAP), 1875 protein-coding sequences were found, 194 of which were assigned to a subsystem according to the annotation conducted by the RAST server. Additionally, 57 RNA genes were identified (Table 2).

Most annotated genes were involved in metabolic functions, such as protein metabolism, carbohydrate metabolism, amino acid anabolism, and the biosynthesis of cofactors or other secondary metabolites. Twenty-one genes encoding putative protein-degrading enzymes belonging to different families of serine (e.g., fervidolysin) and metallopeptidases were annotated. Enzymes involved in the reduction of disulfide bonds were also identified; thus, strain T possesses an arsenal of keratin-degrading enzymes. Moreover, this approach resulted in the identification of known genes predicted to play a role in the resistance to environmental stresses, such as oxidative stress, cold shock (the CSP family), heat shock, and detoxification. In this regard, the organism seemed to possess many defense mechanisms against heavy metals (i.e., copper, cobalt, zinc, and cadmium) and general drugs via a multidrug resistance efflux pump. Two complete 16S rRNA operons were identified and aligned, showing a 100% sequence identity. Three CRISPR arrays, including 19 genes encoding CRISPR-associated proteins, were identified. Although motility has not been reported in fervidobacteria, 56 genes involved in chemotaxis and flagellar motility were identified. The organism did not contain plasmids.

**Figure 5:**
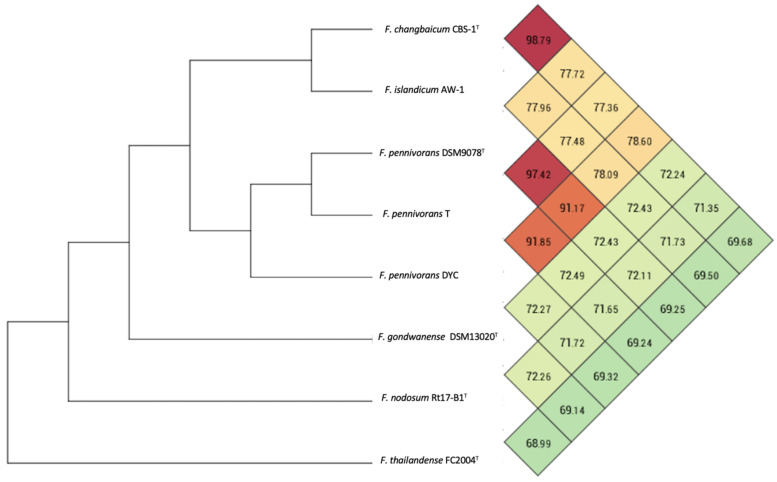
Ortho ANI heatmap and tree calculated with the genome sequences of members of the genus *Fervidobacterium*. The values represent the percentage overall similarity among the different genome sequences. The species cut-off was set at 96%.

A pairwise phylogenomic comparison of *F. pennivorans* strain T against the complete genomes of the members of the genus *Fervidobacterium* was performed using in silico DNA–DNA hybridization (dDDH) analysis using the TYGS server, average nucleotide identity (ANI) calculation, and phylogenomic tree reconstructions. Based on the TYGS analysis, strain T was most closely affiliated with *F. pennivorans* type strain DSM9078, sharing a dDDH identity value of 76.9% (Figure S3), which was consistent with the results of the 16S rRNA-based phylogenetic tree (Figure 2). *F. pennivorans* strain DYC also clustered with strains T and DSM9078, but with dDDH values < 47%, which is significantly below the recommended species threshold value of 70%. This indicates that strain T belongs to the same genome species as that of *F. pennivorans* DSM9078, whereas strain DYC constitutes a separate *Fervidobacterium* species; however, the TYGS analysis indicates that strain T is sufficiently different from DSM9078 to be placed in a separate subspecies cluster, which is also supported by certain metabolic differences, for example, the utilization of cellulose and lactose for growth (Table 2). The other *Fervidobacterium* members formed distinct and separate species clusters, with dDDH values less than 21% compared with strain T and separated by high pseudo-bootstrap values (Figure S3). An Ortho ANI-based phylogenomic tree and heatmap confirmed the TYGS analysis, showing a clustering of strains T, DSM9078, and DYC (Figure 5), with an ANI value of 97.42% between the strains T and DSM9078. Strain DYC shared an ANI value of less than 92% with strains T and DSM9078, supporting the notion that this strain constitutes a separate genome species. Notably, the *F. changbaicum* strain DSM 17883 and *F. islandicum* AW-1 clustered closely together and shared dDDH and ANI values of 88.7% and 98.79%, respectively, strongly suggesting that strain AW-1 belongs to *F. changbaicum* based on the high overall genome sequence similarity. We proposed the name *F. pennivorans* supsp. *keratinolyticus* subsp. nov. for strain T as it is a particularly efficient keratin degrader.

**Figure 6:**
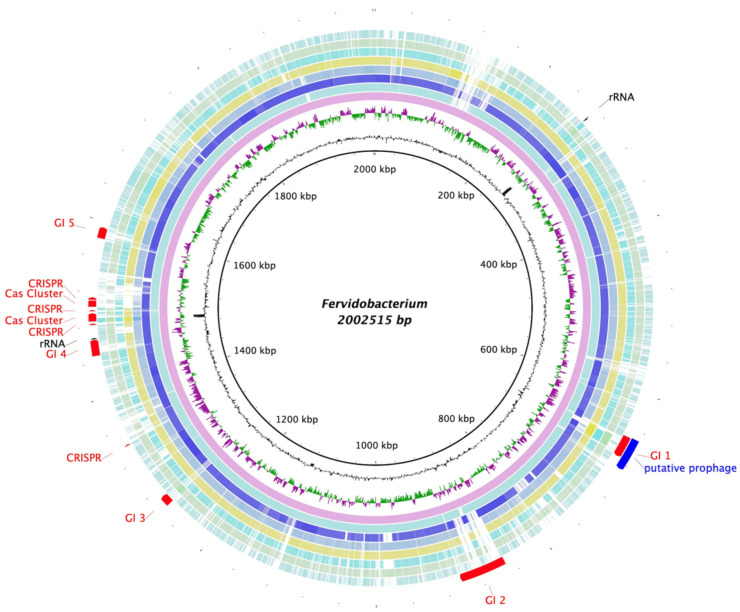
Blast Ring Generator (BRIG) representation of the comparison of the *Fervidobacterium* species’ genomes using strain T as a reference. The innermost circles show strain T’s G + C content (black) and sections with a GC skew (purple/green). The external circles represent the different genomes of the members of *Fervidobacterium* genus, starting with *F. pennivorans* strain T (pink) and followed, from inside to outside, by *F. pennivorans* DSM9078 (pale blue), *F. pennivorans* DYC (navy blue), *F changbaicum* CBS-1 (blue grey), *F. islandicum* AW-1 (yellow), *F. nodosum* Rt17-B1 (turquoise), *F. gondwanense* (green), and *F. thailandense* (sky blue). Finally, the most external area of the figure shows the 16S rRNA genes (black), the predicted genomic islands, CRISPR and Cas9 clusters (red), and a putative prophage, all found in strain T’s genome.

The genomes of representative strains of *Fervidobacterium* spp. were also compared using the BLAST Ring Generator (BRIG) [40], with the genome of strain T as a reference (Figure 6). In addition, the genome of strain T was used as an input for three different tools: the CRISPRCasFinder [41] program was run to detect CRISPR/Cas9 clusters, as shown in Figure 6. Moreover, regions of probable horizontal transfer origin were predicted using the IslandViewer web server [42]. These predicted genomic islands are also displayed in Figure 6, which includes, among other features, a zinc metallopeptidase (QIV78721.1) and nine transposase-related genes. Finally, a region identified as a putative prophage by the PHASTER pipeline [43,44] can also be seen in the same figure, partially overlapped by one of the predicted genomic islands.

**Figure 7:**
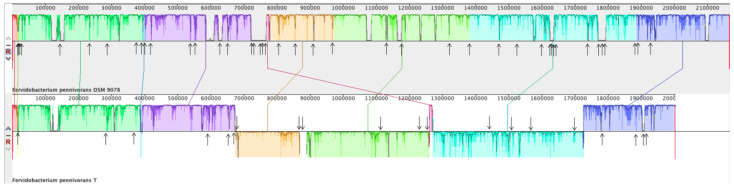
Full genome alignment of *Fervidobacterium pennivorans* DSM9078 (species type strain) and *F. pennivorans* strain T using Mauve. The upper panel represents the DSM9078 genome and the bottom one corresponds to strain T. Fragments with the same color belong to homologous regions, and sequences below the horizontal division correspond to inverted regions. The arrows point to transposase genes, as predicted by genome annotation.

A full genome alignment of *F. pennivorans* strain T and type strain DSM 9078 was performed using Mauve [45] (Figure 7). By annotation with the NCBI Prokaryotic Genome Annotation Pipeline (PGAP), 41 transposases were predicted in DSM 9078 and 21 in strain T. Figure 6 shows this full genome alignment, with the position of the predicted transposases indicated by arrows. In addition, three large genomic inversions were detected when aligning these two genomes, all of which were flanked by transposase-encoding genes.

## 4. Discussion

The new isolate described in this study belongs to the *F. pennivorans* species, although some minor differences between this strain and the species-type strain (DSM9078) could be identified, suggesting that these two bacteria may be classified as different subspecies. The strain grew well on substrates such as glucose, peptone, and galactose; however, turbid cultures were obtained only when yeast extract was added as a supplemental nutrient, suggesting the need for a mixture of the auxiliary nutrients for growth. This behavior is not unique to this organism and has also been described for other strains belonging to the *F. pennivorans* species [46] as well as for many other strict anaerobes. The first batch growth curve for a member of the *F. pennivorans* species was established. It showed a lag phase dependent on both physical and physiological conditions: an exponential phase of 11 h with a generation time of 150 min and a long stationary phase. The total genome size of strain T was 2,002,515 base pairs, similar to those of the other members of the genus *Fervidobacterium*, with a similar number of coding gene sequences. Whole-genome alignment with the type strain showed three large, inverted regions flanked by transposase-encoding genes. Chromosomal rearrangements are not unusual for this group and have also been reported in other Thermotogales species. Chromosomal rearrangements have been suggested to affect species evolution [47,48].

The phylogenetic analyses showed that strain T belongs to the same species group as the type strain (DSM 9078), whereas the DYC strain forms a deeper and separate branch and shares less than a 92% ANI value with strain T and DSM9078, suggesting that strain DYC constitutes a separate *Fervidobacterium* species. However, *F. changbaicum* CB5-1 and *F. islandicum* AW-1 were sufficiently similar to be grouped into the same genome species, with an ANI value of 98.79% (Figure 4). According to the TYGS and OrthoANI analyses, strain T should be considered a separate subspecies. 

Analysis of the genome of *F. pennivorans* strain T allowed the identification of several keratinase enzyme candidates. The strain showed strong keratinolytic activity and could completely degrade chicken feathers after 48 h incubation at 75 °C. Furthermore, scanning electronic microscope images revealed that this bacterium physically attaches to the feathers during the degradation process, suggesting that the cell wall or membrane-bound enzymes participate in the process. Nevertheless, the features required for keratin degradation are not fully understood, and most research in this field centers on screening novel microorganisms with keratinolytic activity, as well as their secreted protease arsenal. Furthermore, for other complex substrates, such as cellulose, it has been suggested that the degradation of keratin does not occur by the action of a single enzyme but requires a mixture of different enzymes with hydrolytic activity. *F. pennivorans* is an anaerobic and thermophilic bacterium, making it especially convenient for industrial processes, as neither oxygen supply nor refrigeration systems are required.

## Figures and Tables

**Table 1:** Characteristics of strain T and all described *Fervidobacterium* type strains.

Characteristic	*F. pennivorans* subsp. *keratinolyticus* T	*F. pennivorans* DSM9078	*F. thailandense* FC2004	*F. nodosum* Rt17-B1	*F. islandicum* H21	*F. gondwanense* AB39	*F. changbaicum* CBS-1
Isolation Source	Tajikistan	Azores Islands, Portugal	Thailand	New Zealand	Iceland	Geothermal artesian basin, Australia	China
Cell Size (µm)	**0.5 × 2–20**	0.5 × 2–20	0.5–0.6 × 1.1–30	0.5–0.55 × 1–2.5	0.6 × 1–4	0.5–0.6 × 4–40	0.5–0.6 × 1–8
Temperature Range (°C) Optimum	**55–75**	50–80	60–88	47–80	50–80	>45 to <80	55–90
	**65**	70	78–80	70	65	65–68	75–80
pH Range Optimum	**6.5–7.5**	5.5–8.0	6.0–8.5	6.0–8.0	6.0–8.0	6.0–8.0	6.3–8.5
	**6.5**	6.5	7.5–8	7	7.2	7	7.5
NaCl Range (g/L) Optimum	**0–30**	0–40	0–5	<10	<10	0–6	0–10
	**3**	4	0–1.0	≤1 (NR)	2	1	0
Generation Time (min)	**150**	126	85	105	150	79	99
Utilization of							
Glucose	**+**	+	+	+	+	+	+
Sucrose	**slow**	**+**	+	+	**+**	**slow**	+
Lactose	**+**	−	−	+	−	+	+
Arabinose	**−**	−	−	slow	+	−	−
Galactose	**+**	+	−	+	+	slow	+
Mannose	**slow**	+	−	+	+	+	−
Sorbitol	**+**	**slow**	−	+	**+**	**−**	+
Mannitol	**−**	**slow**	−	**slow**	**−**	**slow**	−
Starch	**slow**	+	+	+	+	+	+
Cellulose	**+**	−	−	−	+	−	−
Peptone	**+**	+	+	+	+	+	+
Feather Hydrolysis	**+**	+	+	−	−	**−**	−
DNA G + C Content (mol%)	**39**	38.9	45.8	33.7	41	35	31.9

Data from [9]; +: growth after 24 h incubation; −: no growth after 24 h incubation; Slow: growth after 48 h incubation. Greyed in bold: characteristics determined in this work.

**Table 2:** General characteristics of the members of the *Fervidobacterium* genus *.

	*F. pennivorans* T	*F. pennivorans* DSM9078	*F. pennivorans* DYC	*F. islandicum* AW-1	*F. thailandense FC2004*	*F. nodosum* Rt17-B1	*F. changbaicum* CBS-1	*F. gondwanense* 13020
Country of Origin	Tajikistan	Portugal (Azores)	New Zealand	Indonesia	Thailand	New Zealand	China	Australia
Year of Isolation	2021	1999	2016	2004	2016	1985	2007	1996
Genome Size (bps)	2,002,515	2,166,381	2,061,852	2,237,377	2,040,210	1,948,941	2,266,449	2,145,239
N° CDS with Protein	1875	1973	1893	2055	1870	1796	2038	1975
N° RNAs	57	57	58	56	54	58	57	54
N° rRNAs (23S-16S-5S)	2 Feburary 2002	3, 1, 2	2, 2, 2	2, 2, 2	1, 3, 1	2, 2, 2	2, 2, 2	1, 3, 1
% GC	39	38.9	38.9	40.7	45.8	3	40.7	39.7
CRISPR Clusters	3	4	3	2	8	2	2	1
NCBI Accession Number	CP050868	CP003260.1	CP011393.1	CP014334.2	NZ_LWAF00000000.1	CP000771.1	NZ_CP026721.1	NZ_FRDJ00000000.1

* The data were retrieved from Genbank [9].

## Data Availability

The complete genome sequence is available under GenBank accession number CP050868.

## Supplementary Materials

The following are available online at https://www.mdpi.com/article/10.3390/microorganisms11010022/s1: Figure S1 (Phylogenetic tree based on the 16S rRNA gene inferred by the Maximum Likelihood method), Figure S2 (Growth curve of *F. pennivorans* subsp. *keratinolyticus* strain T in anaerobic MMF medium supplemented with 0.1 % yeast extract and 0.5 % glucose) and Figure S3 (Phylogenomic tree of *F. pennivorans* subsp. *keratinolyticus* strain T and related *Fervidobacterium* species and strains constructed by using the TYGS genome server (https://tygs.dsmz.de, accessed on 11 October 2022)).
