# Supplementary material for: Fervidobacterium pennivorans subsp. keratinolyticus subsp. nov., a Novel Feather-Degrading Anaerobic Thermophile
Source: Microorganisms. 2022 Dec 21;11(1):22. doi: 10.3390/microorganisms11010022 (PMC9861569; doi:10.3390/microorganisms11010022)
Supplement: Supplementary file 1 [file microorganisms-11-00022-s001.zip › microorganisms-2088681-supplementary.pdf]

SUPPLEMENTARY MATERIAL

Fervidobacterium pennivorans subsp. *keratinolyticus* subsp. nov., a novel feather-degrading anaerobic thermophile

Ruben Javier-Lopez, Edoardo Mandolini, Munavvara Dzhuraeva, Khursheda Bobodzhanova and Nils-Kåre Birkeland

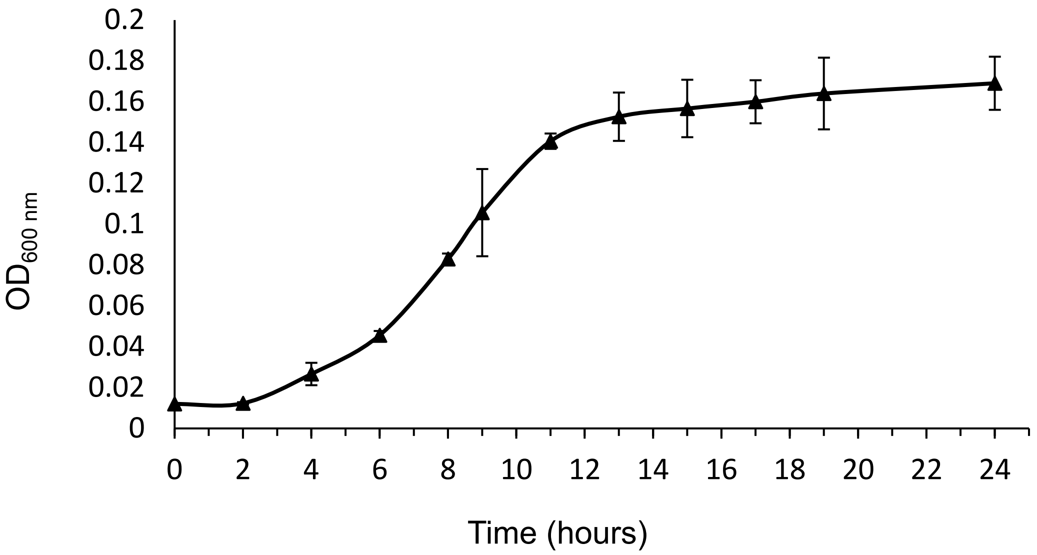

Figure S1. Growth curve of *F. pennivorans* subsp. *keratinolyticus* strain T in anaerobic MMF medium supplemented with 0.1 % yeast extract and 0.5 % glucose and incubated statically with occasional shaking at 65 ºC. The indicted values including standard deviations are based on average of three biological replicates.

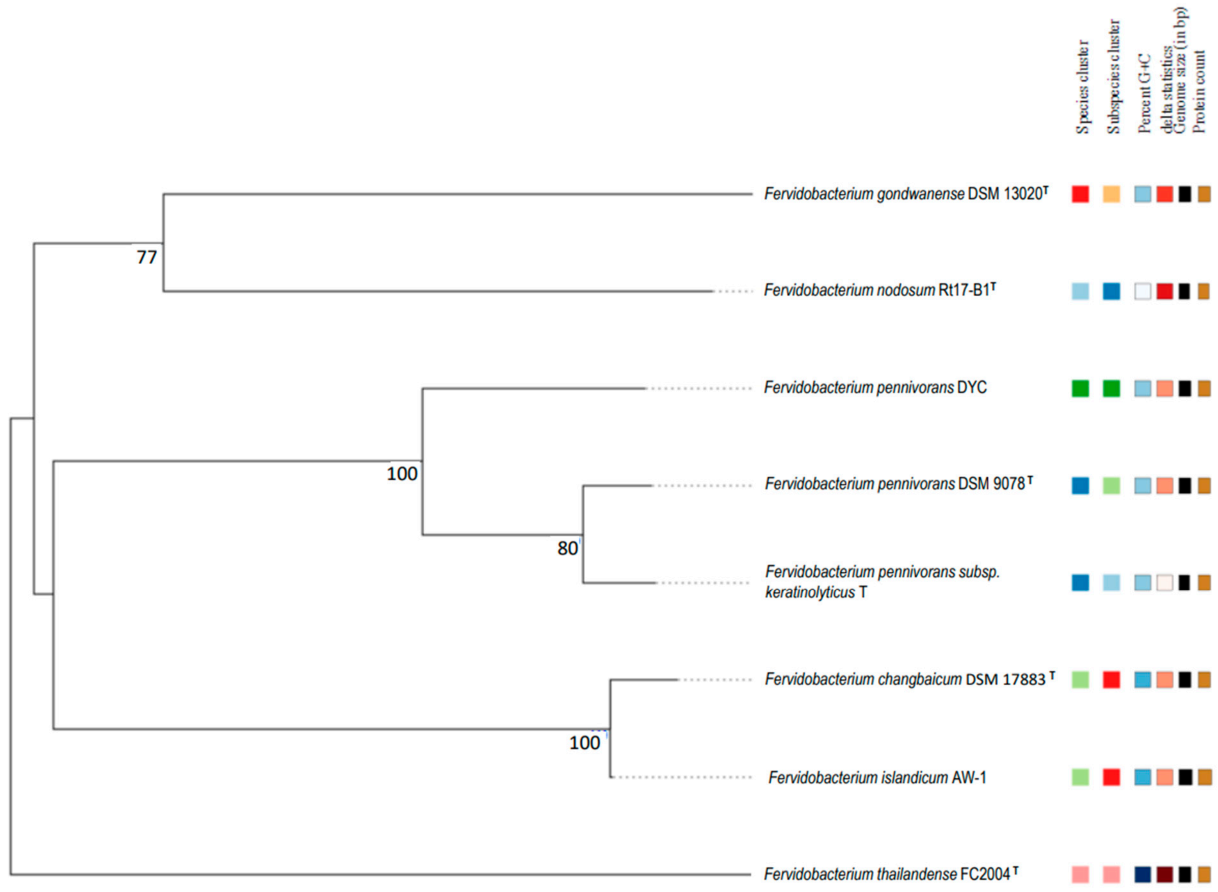

Figure. S2. Phylogenomic tree of *F. pennivorans* subsp. *keratinolyticus* strain T and related *Fervidobacterium* species and strains constructed by using the TYGS genome server (https://tygs.dsmz.de). The tree was inferred with FastME 2.1.6.1 from GBDP distances calculated from genome sequences, with an average branch support of 77.2%. The branch lengths are scaled in terms of GBDP distance formula d5. The numbers at branches are GBDP pseudo-bootstrap support values ≥77% from 100 replications. The tree was rooted at midpoint. Genome sequence accession numbers: *F. gondwanense*, GCA_900143265; *F. nodosum*, GCA_000017545; *F. pennivorans* strain DYC, GCA_001644665.1; *F. pennivorans* DSM 9078, GCA_000235405; *F. pennivorans* subsp. *keratinolyticus* strain T, CP050868; *F. changbaicum*, GCA_900100515; *F. islandicum* AW-1, GCA_000767275.4; *F. thailandense*, GCA_001719065.
